# Lifestyle Intervention for the Prevention of Diabetes in Women With Previous Gestational Diabetes Mellitus: A Systematic Review and Meta-Analysis

**DOI:** 10.3389/fendo.2018.00583

**Published:** 2018-10-05

**Authors:** Pâmella Goveia, Wilson Cañon-Montañez, Danilo de Paula Santos, Gabriela W. Lopes, Ronald C. W. Ma, Bruce B. Duncan, Patricia K. Ziegelman, Maria Inês Schmidt

**Affiliations:** ^1^Postgraduate Program in Epidemiology, Universidade Federal do Rio Grande do Sul, Porto Alegre, Brazil; ^2^Faculty of Nursing, Universidad de Antioquia, Medellín, Colombia; ^3^Department of Medicine and Therapeutics, The Chinese University of Hong Kong, Prince of Wales Hospital, Shatin, China

**Keywords:** diabetes, gestational, diabetes mellitus, life style, primary prevention, women

## Abstract

**Background:** Type 2 diabetes is increasing among the young, and gestational diabetes (GDM) offers a unique opportunity for diabetes prevention. We aimed to systematically review postpartum randomized trials to summarize the benefits of lifestyle interventions for women with previous GDM.

**Methods:**We searched for RCTs involving women with previous GDM that compared lifestyle interventions—diet, physical activity or breastfeeding—at postpartum with usual care up to May 2018.

**Results:**Of 1,895 abstracts identified, we selected 15 studies investigating incidence of diabetes or changes in glycemia. Most interventions focused on changes in diet and physical activity, only one also on incentive to breastfeeding. Meta-analysis of 8 studies investigating incidence of diabetes revealed a homogeneous (I^2^ = 10%), reduction of 25% (RR = 0.75; 95%CI: 0.55–1.03) borderline statistically significant. Only trials offering intervention soon after delivery (< 6 months post-partum) were effective (RR = 0.61; 95%CI: 0.40–0.94; p for subgroup comparison = 0.11). Overall, no benefit was found regarding measures of glycemia. Although moderate reductions in weight (MD = −1.07 kg; −1.43−0.72 kg); BMI (MD = −0.94 kg/m^2^; −1.79 −0.09 kg/m^2^); and waist circumference (MD = −0.98 cm; −1.75 −0.21 cm) were observed, effects were larger with longer follow-up.

**Conclusions:**Summary results of the available evidence support benefits of lifestyle interventions at postpartum for women with previous GDM. Benefits, although smaller than those of major trials based in older subjects receiving intensive interventions, appear clinically relevant for this young subset of woman. Further studies are needed to improve the quality of the evidence and to further tailor interventions to this specific setting.

## Introduction

The International Diabetes Federation (IDF) estimates that at least 425 million persons in the world have diabetes (1).From 1980 to 2014 the global age–standardized prevalence of diabetes in adults more than doubled in men and increased almost 60% in women (2). If these trends continue, the World Health Organization (WHO) goal of halting the rise of diabetes by 2025 will not be achieved (2). The increasing burden of diabetes challenges individuals, families and health systems globally.

Diabetes can be prevented or delayed with intensive lifestyle changes offered to high-risk people, as indicated as indicated by the following now classical studies. The Da Qing Diabetes Prevention Study, after 6 years of lifestyle intervention, reduced the incidence of diabetes by 31, 46, and 42% in the groups of diet, exercise and diet plus exercise, respectively (3),and benefits extended over 20 years after the intervention was discontinued (4). The Finnish Diabetes Prevention Study (DPS) and the Diabetes Prevention Program (DPP) both showed a reduction of 58% in the incidence of diabetes mellitus in individuals with impaired glucose tolerance after an average of 3 years of lifestyle interventions focusing on diet and physical activity (5, 6). A recent systematic review of 43 studies evaluating the long-term sustainability of diabetes prevention approaches showed that the superiority of lifestyle interventions over medications observed at the end of the trial persisted for many years (7). The review included 49,029 participants with mean age of 57.3 (±8.7) years, indicating that the younger age group has been little evaluated.

Of great concern, prevalence of type 2 diabetes is increasing among the young, a phenomenon potentially increasing the burden of disease owing to the longer duration of diabetes and the apparently high incidence of chronic complications of those so affected (8, 9). Thus, diabetes prevention starting earlier than the settings of most published trials is of paramount importance. Gestational diabetes mellitus (GDM) offers a unique opportunity for diabetes prevention in younger adults. First, the diagnosis of GDM confers an increased risk of diabetes and its complications which appears to be mediated at least in part by subsequent weight gain and lack of a healthy lifestyle (10). Initial studies testing the efficacy of lifestyle interventions suggest benefit (11–25), but few systematic reviews have been carried out so far (26–28), with only one attempting to assess diabetes as an outcome (26).

We aim to systematically review and summarize the benefits of lifestyle interventions in the prevention of diabetes as well as in reduction of plasma glucose levels and anthropometry measures in women with recent GDM, as evaluated in postpartum randomized controlled trials.

## Methods

### Protocol and registration

This is a systematic review and meta-analysis of randomized controlled trials (RCTs), registered with the International Prospective Register of Ongoing Systematic Reviews (PROSPERO) under the number CRD42018092440, and following the recommendations of the Preferred Reporting Items for Systematic Reviews and Meta-Analyses (PRISMA Statement) (29) and the Cochrane Handbook for Systematic Reviews of Interventions.

### Eligibility criteria

The review included all RCTs involving women with previous GDM (as defined by any recognized diagnostic criteria) that compared lifestyle interventions—diet and/or physical activity and/or breastfeeding—with usual care without pharmacological treatment. We included only trials assessing the incident of diabetes mellitus (primary outcome) or glycemic levels ((mean change from baseline of fasting or 2 h glucose, or HbA_1C_), our surrogate outcomes. We excluded studies including women with current or previous diagnosis of type 1 or type 2 diabetes, using pharmacological interventions or having recruitment strategies that were not based on a recent diagnosis of GDM.

### Literature search

We searched PubMed, Cochrane Central Register of Controlled Trials, Web of Science and EMBASE databases in May, 2018. The search string for PubMed was: (“Diabetes, Gestational” [Mesh] OR “Diabetes, Pregnancy-Induced” OR “Diabetes, Pregnancy Induced” OR “Pregnancy-Induced Diabetes” OR “Gestational Diabetes” OR “Diabetes Mellitus, Gestational” OR “Gestational Diabetes Mellitus”) AND (“Exercise”[Mesh] OR Exercises OR “Physical Activity” OR “Activities, Physical” OR “Activity, Physical” OR “Physical Activities” OR “Exercise, Physical” OR “Exercises, Physical” OR “Physical Exercise” OR “Physical Exercises” OR “Diet”[Mesh] OR Diets OR “Body Weight”[Mesh] OR “Weight, Body” OR “Weight Loss”[Mesh] OR “Loss, Weight” OR “Losses, Weight” OR “Weight Losses” OR “Weight Reduction” OR “Reduction, Weight” OR “Reductions, Weight” OR “Weight Reductions” OR “Life Style”[Mesh] OR “Life Styles” OR Lifestyle OR Lifestyles) AND (“controlled study” OR trial^*^).These terms were adjusted to fit the requirements of each electronic database. We screened the list of references of the included studies and of systematic reviews to check for other possible studies to be included.

We did not include terms for the primary outcome to enhance the search sensitivity. We made no restrictions regarding language or publication date.

### Data extraction

Initially, two reviewers (DS, GL) independently analyzed titles and abstracts of each paper retrieved to identify potential eligible studies. Inconsistencies were discussed and studies not clearly meeting the inclusion criteria were excluded. Disagreements were resolved by discussion with a third reviewer (PG) whenever necessary.

A standard data form was used to extract the following information: study population, demographic data and baseline characteristics of participants, details of the intervention and the control counterpart, results, moments of measurement; and information for assessment of risk of bias.

Relevant missing information was requested from the original authors. Procedures for estimation of missing data were performed whenever possible (29). If data were still insufficient after these processes, the outcome was included in descriptive analysis only.

### Outcomes

The primary outcome was incidence of diabetes mellitus. We also reported change in glycemic levels (mean fasting or 2 h glucose, or HbA_1C_). Secondary outcomes were changes in the anthropometric measures of weight and waist circumference.

### Risk of bias (quality) assessment

Three reviewers in pairs (DS, GL, PG) independently assessed the quality of the studies. The disagreements were resolved by consensus or with the consultation of an additional author (WC).

We evaluated the risk of bias as described in the Cochrane Handbook for Systematic Reviews of Interventions using the Cochrane Collaboration tool (29), with the following criteria: random sequence generation (selection bias); allocation concealment (selection bias); blinding (performance bias and detection bias) considering blinding of participants, personnel and those performing outcome assessment; incomplete outcome data (attrition bias); selective reporting (reporting bias); and other biases.

### Data analysis

We estimated relative risks for the incidence of diabetes mellitus. For continuous outcomes, we estimated mean differences from baseline. When standard deviations for changes were missing, we made imputations considering a conservator correlation equal to zero. We used random effects models with DerSimonian and Laird estimators for analyses of all outcomes. All statistical tests were two-sided and significance was defined as *P* < 0.05. We assessed statistical heterogeneity of treatment effects across studies using the I^2^ metric statistics. The statistical analyses were performed used R version 3.5.0 (R Foundation for Statistical Computing). In addition, publication bias was examined using funnel plot and the Egger test (Stata 11.0, StataCorp, College Station, TX).

## Results

### Study selection and patient characteristics

**Figure 1:**
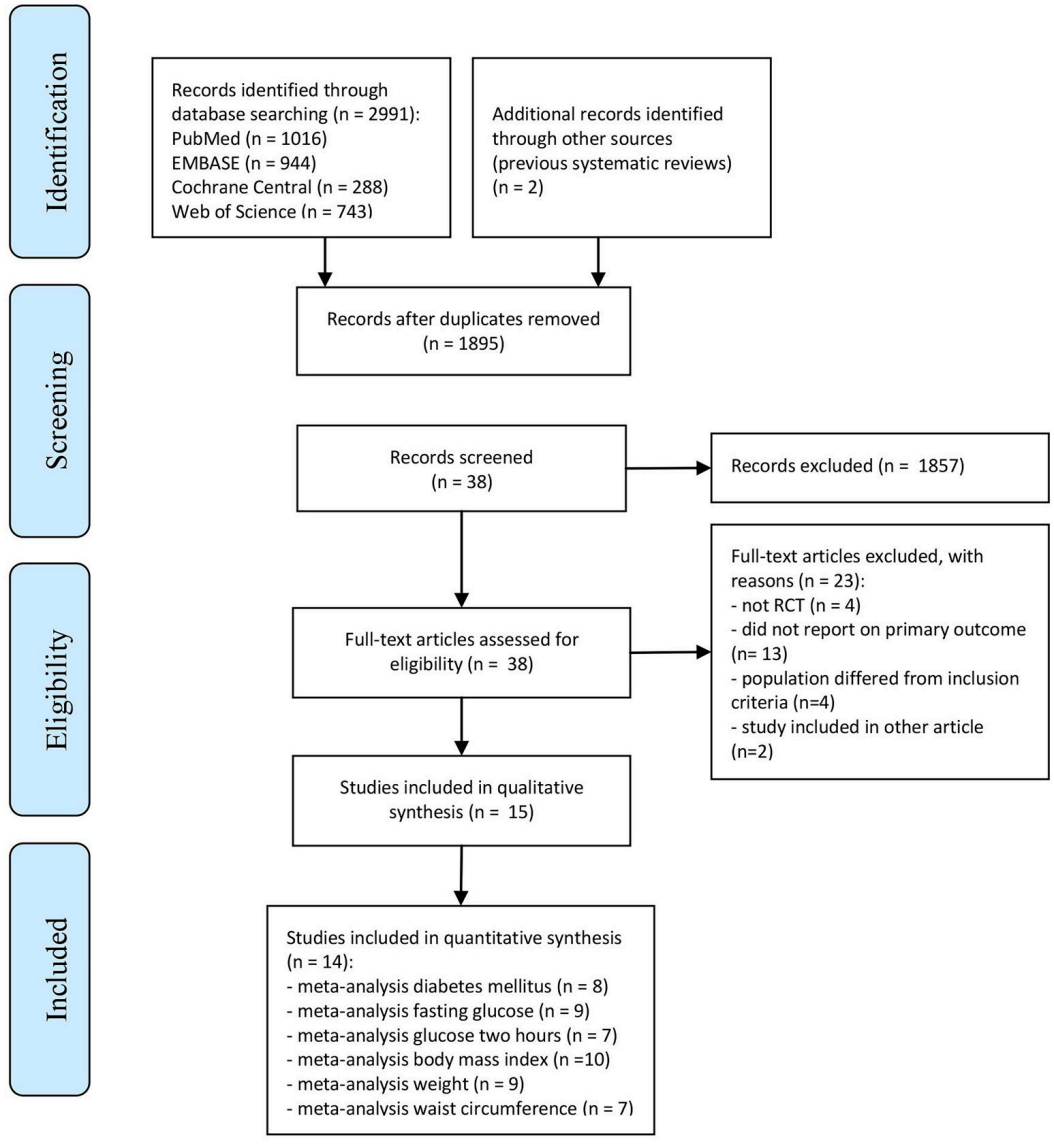
Flow chart summarizing the process for the identification of the eligible studies.

The flowchart for the selection and exclusion of studies is presented in Figure 1. After removing duplicates, we found a total of 1,895 abstracts from where 38 articles were considered as potentially eligible and assessed through full-text reading. We then excluded 23 additional studies, remaining with a total of 15 studies. The reasons for exclusion were: not a randomized controlled trial (*n* = 4) (30–33), not reporting our primary outcomes (*n* = 13) (34–46), study population not meeting our inclusion criteria specification (*n* = 4) (47–50) and different reports from the same study (*n* = 2) (51, 52).

The 15 studies included in the systematic review are described in Table 1. All articles were published within the last 10 years, except one (11). Studies took place in the United States (11–13), Australia (14–16, 25), China (17–20), Spain (21), Malaysia (22), Israel (23), and Ireland (24). The number of women who were randomized in individual studies ranged from 28 to 573, with 8 studies including at least 100 participants (11, 16–21, 23). Ten studies specified eligibility criteria regarding the risk of diabetes: postpartum glucose intolerance (11, 17, 18, 20–22, 24), overweight or obesity (13, 22, 24), low level of physical activity (12, 25), altered lipid profile (24), high waist circumference (22, 24), family history of diabetes (22), use of insulin during pregnancy (17) or hypertension (24).

**Table 1:** Characteristics of the included studies.

**First author and year**	**Country**	**Sample size (randomized)**	**Period of intervention following partum: start point; endpoint**.	**Inclusion criterion in addition to recent GDM**	**Intervention duration/Follow-up**	**Focus of interventions**	**Mode of interventions**	**Outcome measures**
Cheung et al. (25)	Australia	43	Not specified; 4 years	Sedentary habits	1 year	Exercise	1 individual meeting (clinic visit) pedometer 5 telephone contacts 7 postcards	DM BMI
Hu et al. (19)	China	444	1 year; 6 years	–	1 year (in this article; total follow-up 2 years)	Diet and exercise	6 individual meetings (clinic visits)	DM FBG OGTT HbA_1C_ BMI WC
Ji et al. (18)	China	144	Shortly after partum; not specified.	Postpartum IGT or IFG	4 months	Diet and exercise	•4 individual meetings (home visits) •Diary (diet, exercise, weight, postprandial blood glucose) •3 telephone contacts	DM FBG OGTT HbA_1C_ BMI Weight
Kim et al. (12)	United States	49	Not specified; 3 years	Sedentary habits	3 months	Exercise	Web content Internet forum Pedometer Text messages	FBG OGTT BMI Weight WC
McIntrye et al. (15)	Australia	28	Shortly after partum; not specified.	–	3 months	Exercise	1 individual meeting (clinic visit) 7 telephone contacts	FBG Weight WC
Nicklas et al. (13)	United States	75	Shortly after partum; not specified.	BMI > 22/24 kg/m^2^ and < 50 kg/m^2^	1 year	Diet and exercise; breastfeeding	12 web modules Lifestyle coach available by phone or email Support material (laptop, scale, measuring cups and spoons, membership to the gym)	DM BMI Weight
O'Dea et al. (24)	Ireland	50	1year post partum; 3 years	IGT, IFG or insulin resistance (HOMA) + 2 of the following: hypertension, high total cholesterol, triglycerides or LDL-C, low HDL-C, BMI >30 kg/m^2^, WC>88 cm.	12–16 weeks of intervention/1year of follow-up	Diet and exercise	1 individual meeting (clinic visit) 12 group sessions with individual meeting at the end of each group session	FBG OGTT BMI Weight WC
O'Reilly et al. (16)	Australia	573	Notspecified; 1 year	–	1 year	Diet and exercise	1 individual meeting (home visit) 5 group sessions 2 telephone contacts	FBG BMI Weight WC
Peacock et al. (14)	Australia	31	6 months after partum; 2years	–	3 months	Diet and exercise	4 nutrition coaching workshops Pedometer Text messages if the participant uploaded accelerometry data	FBG BMI Weight WC
Pérez-Ferre et al. (21)	Spain	260	Shortly after partum; not specified.	Exclusion of postpartum IFG	3 years	Diet and exercise	Training exercise (at the hospital, 20 training sessions in 10 weeks) 4 individual meetings (clinic visits)	DM FBG HbA_1C_ BMI WC
Shek et al. (17)	China	450	Shortly after partum; not specified.	Postpartum IGT; exclusion if insulin use during pregnancy	3 years	Diet and exercise	7 individual meetings (clinic visits) Diary (diet and exercise) of 5 days prior to each visit	DM BMI
Shyam et al. (22)	Malaysia	77	Shortly after partum; not specified.	Family history of diabetes and: BMI >23 kg/m^2^ or WC 80 cm or IGT or IFG	6 months	Diet	1 individual meeting (clinical visit)	FBG OGTT BMI Weight WC
Wein et al. (11)	United States	200	Shortly after partum; not specified.	Postpartum IGT	7.1 to 81 months (median of 51months)	Diet and exercise	3 monthly telephone contacts	DM FBG OGTT BMI
Yu et al. (20)	China	126	Shortly after partum; not specified.	Postpartum IGT/IFG	2 years	Diet and exercise	4 individual meetings (clinic visits) 4 telephone contacts	DM BMI
Zilberman-Kravits et al. (23)	Israel	180	3–4 months after partum; not specified.	–	2 years	Diet and exercise	3 individual meetings (clinic visits) 3 to 4 group sessions	FBG BMI Weight WC

*IFG, impaired fasting glucose; IGT, impaired glucose tolerance; DM, diabetes mellitus; FBG, fasting blood glucose; OGTT, standard 2 h oral glucose tolerance test; HbA_1C_, glycated hemoglobin; BMI, body mass index; WC, waist circumference; GDM, gestational diabetes mellitus; LDL-C, low-density lipoprotein cholesterol; HDL-C, high-density lipoprotein cholesterol*.

Duration of follow-up was 6 months or less in 5 studies (12, 14, 15, 18, 22), 1 year in 5 studies (13, 16, 19, 24, 25), and 2 years or more in 5 studies (11, 17, 20, 21, 23).

Most of the interventions focused on changes in diet and physical activity. Only one study mentioned incentive to breastfeed (13). Three studies focused solely on the effectiveness of physical activity intervention (12, 15, 25) and one only on diet (22). Standard/brief advice on diet and/or exercise was considered to be comparable with usual care and accepted as the control comparison. Different ways of delivering the intervention were applied: Nine established remote contact (11–16, 18, 20, 25) (by phone, internet or postcards); four performed group sessions (14, 16, 23, 24), and eleven had individual face-to-face contacts (15–25). From those which held individual meetings, two conducted home visits (16, 18) and the others held the sessions in the clinic/hospital.

Eight trials had data to estimate incident diabetes (11, 13, 17–21, 25). Eleven trials measured glycemic control (11, 12, 14–16, 18, 19, 21–24), and all trials investigated the effect on body weight. Overall, considerable heterogeneity was evident between studies in relation to several key characteristics, namely, the method of the intervention, the time lag since the pregnancy complicated by GDM, the degree of risk beyond having GDM, and the duration of follow-up.

### Quality assessment of included studies

Table 2 presents items necessary to assess risk of bias in each study according to the Cochrane Collaboration risk of bias tool for RCTs. Considering all studies included, 60% described adequate random sequence generation (12–18, 22, 24) and 40% allocation concealment (13–16, 22, 24). We did not evaluate blinding of staff performing the interventions due to the nature of lifestyle interventions. Only 26% of the studies mentioned blinding of the outcome assessors (12–14, 22), and it was frequently unclear whether blinding extended to all staff involved (laboratory technicians, staff making anthropometric assessments, data analysts). About half of the studies described exclusions and losses during follow-up (12–14, 16, 17, 21, 22) and a similar proportion reported intention-to-treat analysis (13, 14, 16, 17, 21, 22, 24). Some studies (11, 19, 20) though not mentioning intention to treat analysis or reasons for losses or exclusions, presented few such events, thus minimizing the possibility of bias due to incomplete outcome data.

**Table 2:** Risk of bias among included studies.

	**Adequate random sequence generation**	**Allocation concealment**	**Blinding of outcome assessment**	**Description of losses and exclusions**	**Intention-to-treat analysis**	**Free from selective reporting**
Cheung et al. (25)	Not informed	Not informed	Not informed	No	No	Unclear^d^
Hu et al. (19)	Not informed	Not informed	Not informed	No	No	Yes
Ji et al. (18)	Yes	Not informed	Not informed	No	No	Unclear^d^
Kim et al. (12)	Yes	Not informed	Yes^a^	Yes	No	No
McIntrye et al. (15)	Yes	Yes	Not informed	No	No	Yes
Nicklas et al. (13)	Yes	Yes	Yes^b^	Yes	Yes	Yes
O'Dea et al. (24)	Yes	Yes	Not informed	No	Yes	Unclear^d^
O'Reilly et al. (16)	Yes	Yes	Not informed	Yes	Yes	Yes
Peacock et al. (14)	Yes	Yes	Yes^b^	Yes	Yes	Yes
Pérez-Ferre et al. (21)	Not informed	Not informed	Not informed	Yes	Yes	Yes
Shek et al. (17)	Yes	Not informed	Not informed	Yes	Yes	Unclear^d^
Shyam et al. (22)	Yes	Yes	Yes^a^^c^	Yes	Yes	Yes
Wein et al. (11)	Not informed	Not informed	Not informed	No	No	Unclear^d^
Yu et al. (20)	Not informed	Not informed	Not informed	No	No	Unclear^d^
Zilberman-Kravits et al. (23)	No	Not informed	Not informed	No	No	Yes

a *Blinding of staff obtaining anthropometry*.

b *Blinding of data analysts*.

c *Blinding of laboratory technicians*.

d *Study registration or published protocol not found*.

### Main results

**Figure 2:**
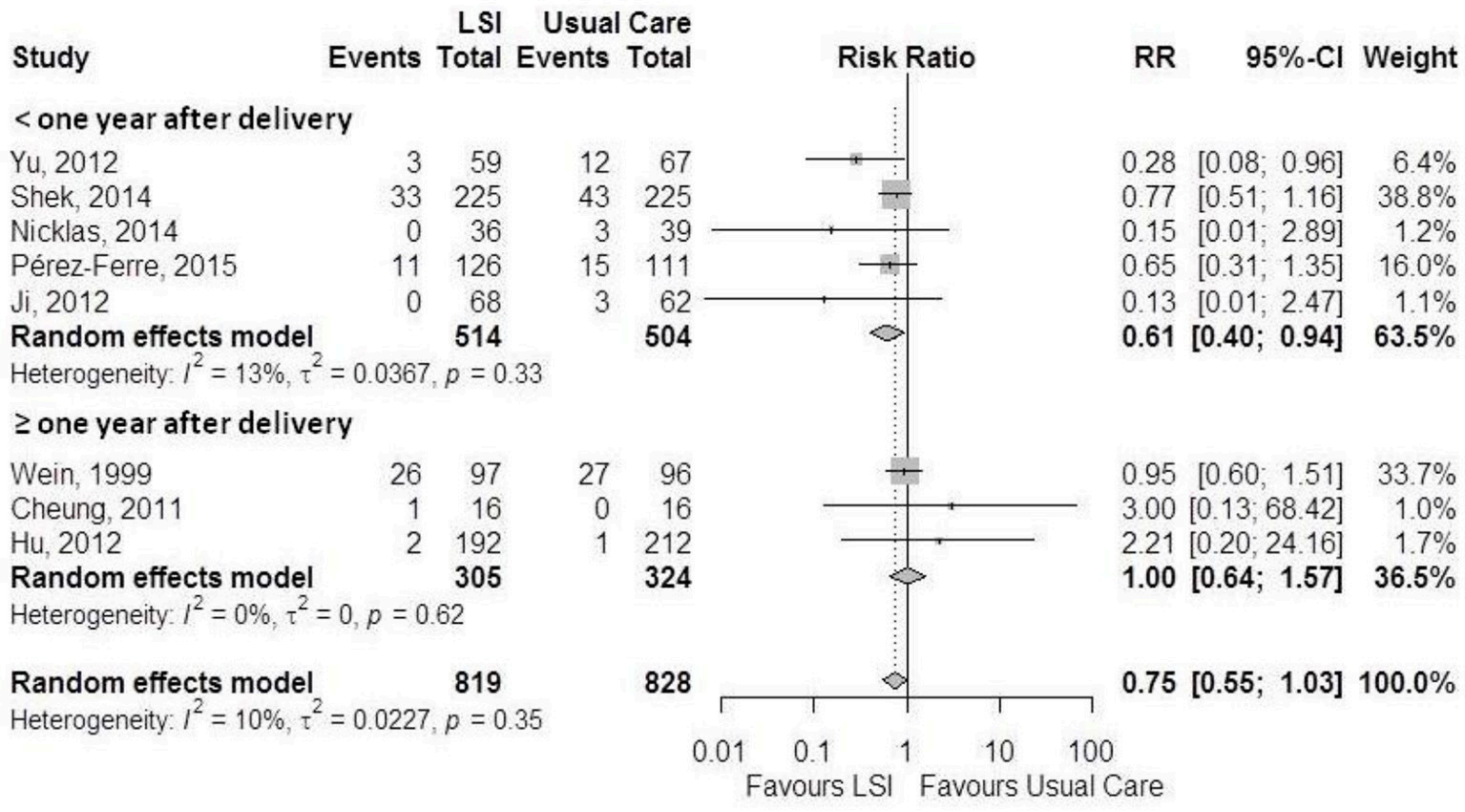
Meta-analysis of the effects of lifestyle interventions (LSI) in diabetes incidence according to post-partum time at randomization.

Meta-analysis of the 8 studies reporting incident diabetes (Figure 2) revealed a borderline statistically significant relative reduction of 25% (RR = 0.75; 95%CI: 0.55–1.03) in incidence with intervention. The results were homogeneous across studies (I^2^ = 10%). When stratified by time of randomization, only studies initiating earlier in the post-partum period showed a significant reduction (RR = 0.61; 95%CI: 0.40–0.94; p for subgroup comparison = 0.33). The overall absolute difference in incidence between groups was −0.04 (95%CI: −0.09; 0.01).

**Figure 3:**
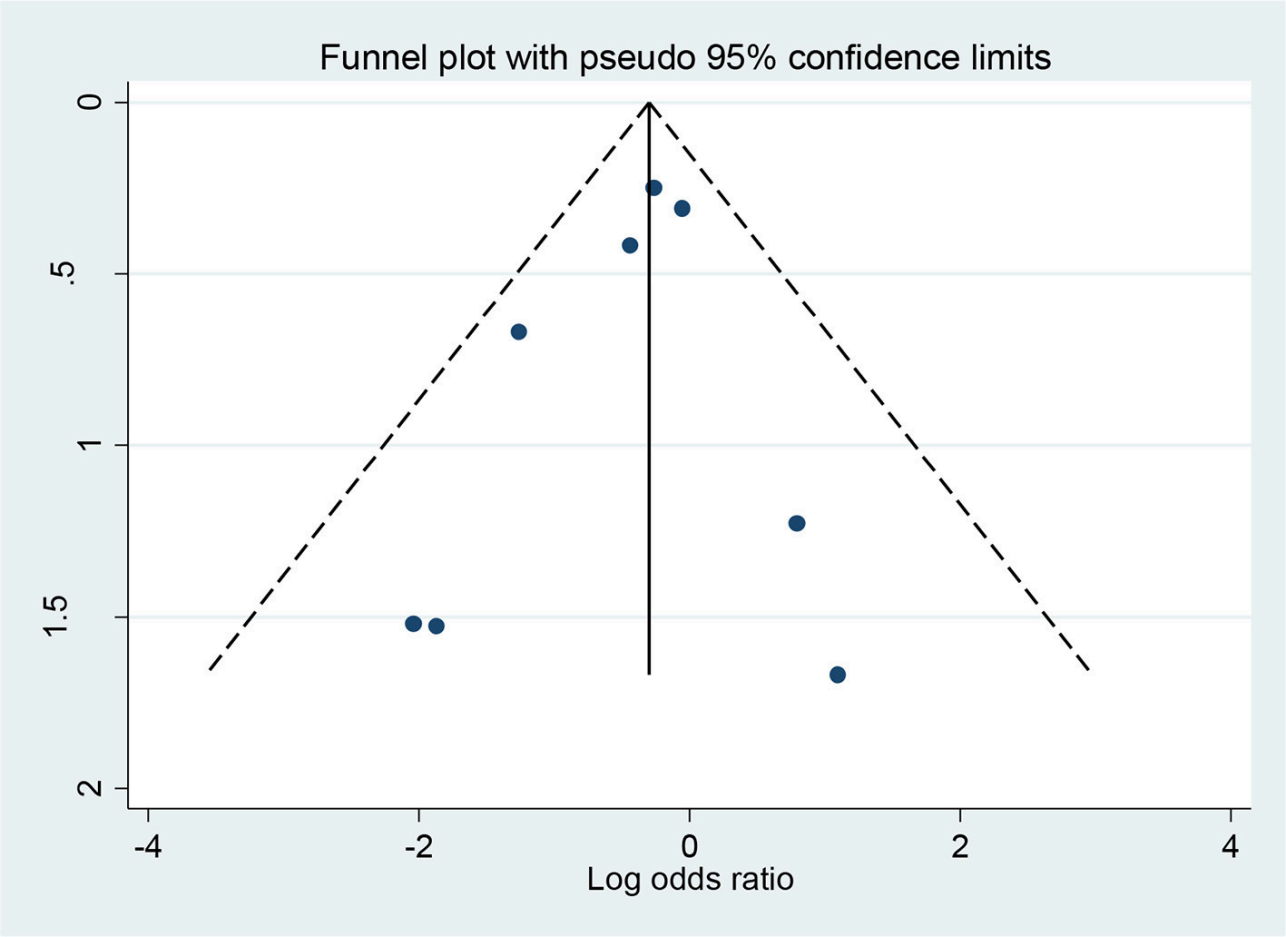
Funnel plot, using data from 8 trials with information for diabetes incidence. Log-odds ratios all displayed on the horizontal axis.

Figure 3 shows a funnel plot for the 8 studies reporting incidence of diabetes. We can observe a general funnel shape indicating that studies of lower precision were spread evenly on both sides of the average, suggesting absence of publication bias. The Egger test also indicated absence of publication bias (*p* = 0.47).

**Figure 4:**
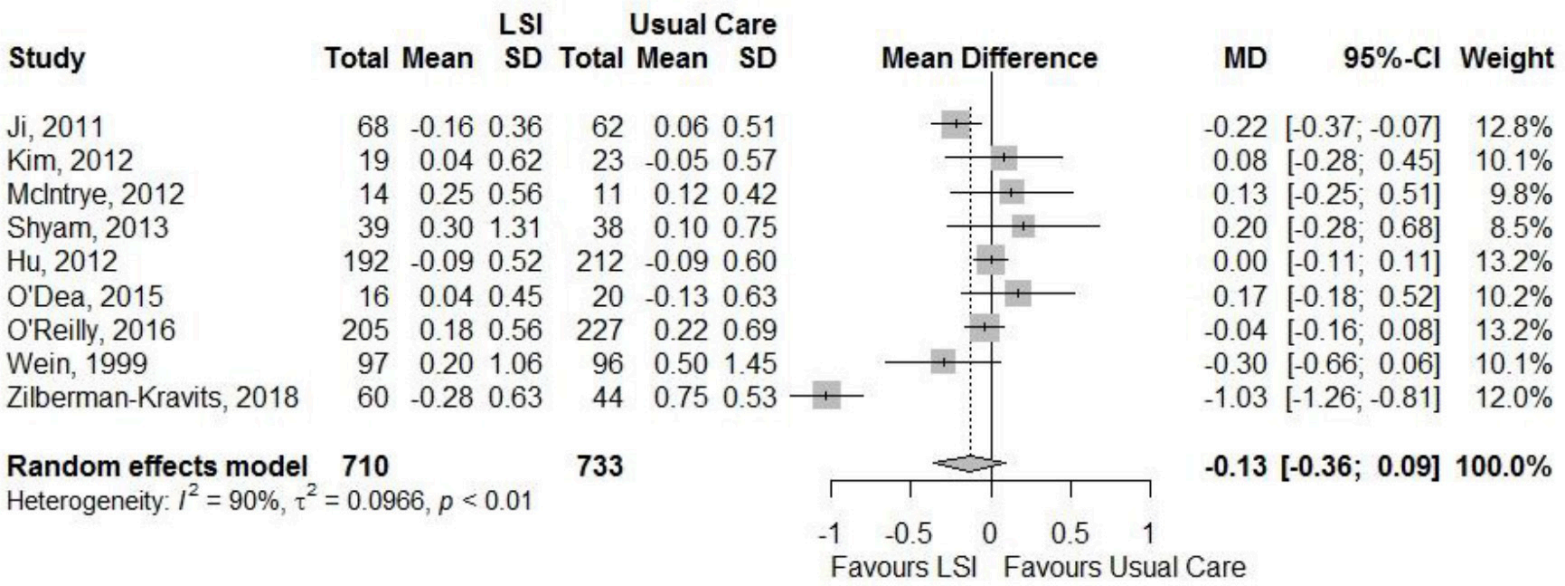
Meta-analysis of the effects of lifestyle interventions (LSI) in fasting glucose change (mmol/L) from baseline to the end of follow up.

**Figure 5:**
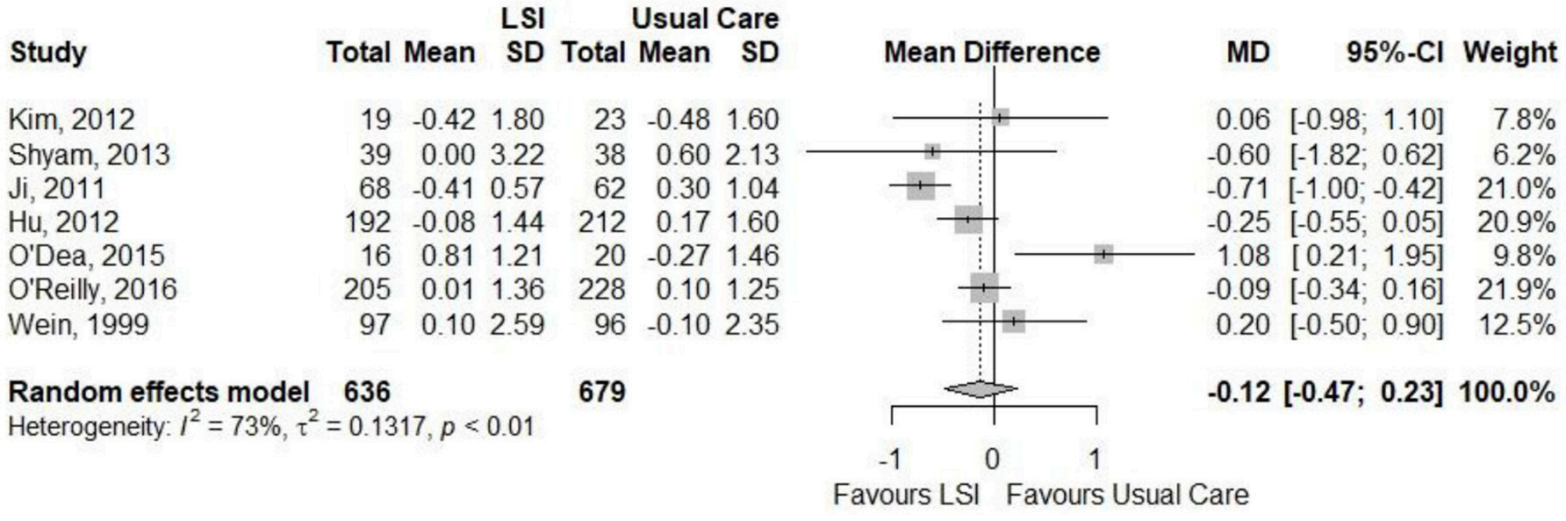
Meta-analysis of the effects of lifestyle interventions (LSI) in 2-h glucose change (mmol/L) from baseline to the end of follow up.

Figures 4, 5 showed a lack of effect of lifestyle interventions in mean fasting and 2h plasma glucose, with a non-significant difference from baseline on fasting glucose (MD = −0.13; 95%CI: −0.36; 0.09) mmol/L and on 2 h glucose (MD = −0.12; 95%CI: 0.47; 0.23) mmol/L for 2 h glucose. Only 3 studies reported HbA1c, without positive results.

**Figure 6:**
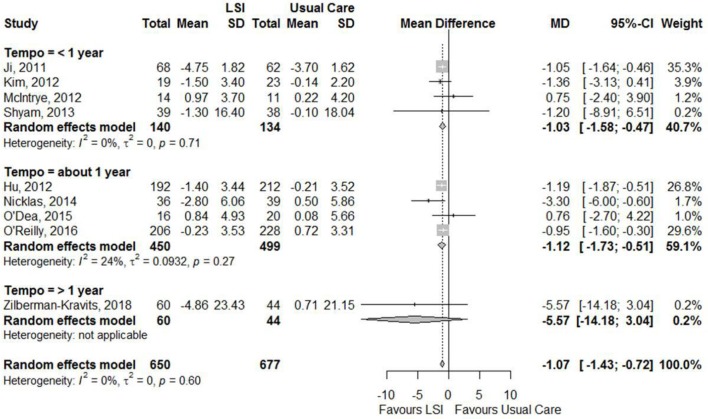
Meta-analysis of the effects of lifestyle interventions (LSI) in weight change (kg) from baseline to the end of follow up, according to the duration of follow-up.

**Figure 7:**
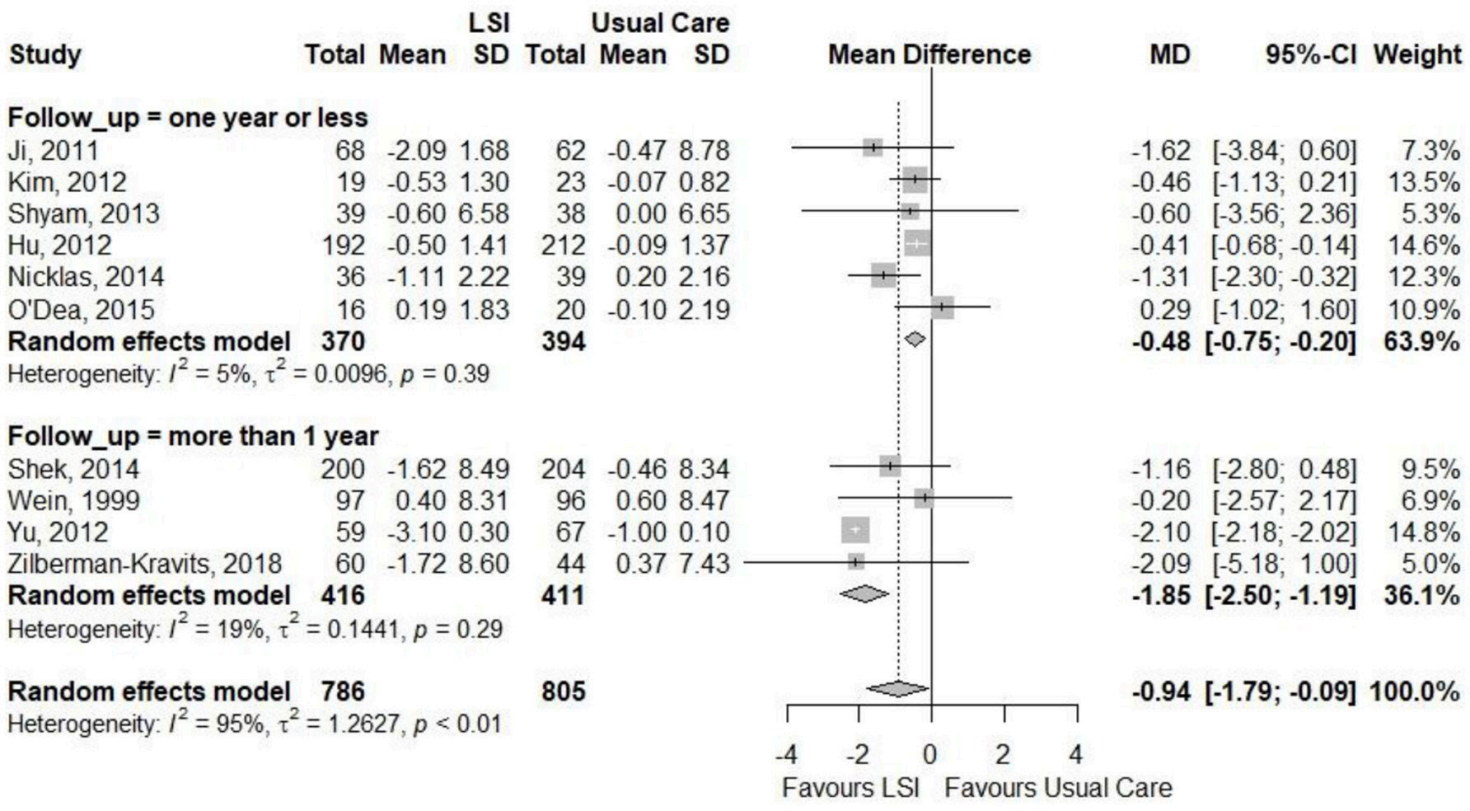
Meta-analysis of the effects of lifestyle interventions (LSI) in BMI change (kg/m2) from baseline to the end of follow up, according to the duration of follow-up.

**Figure 8:**
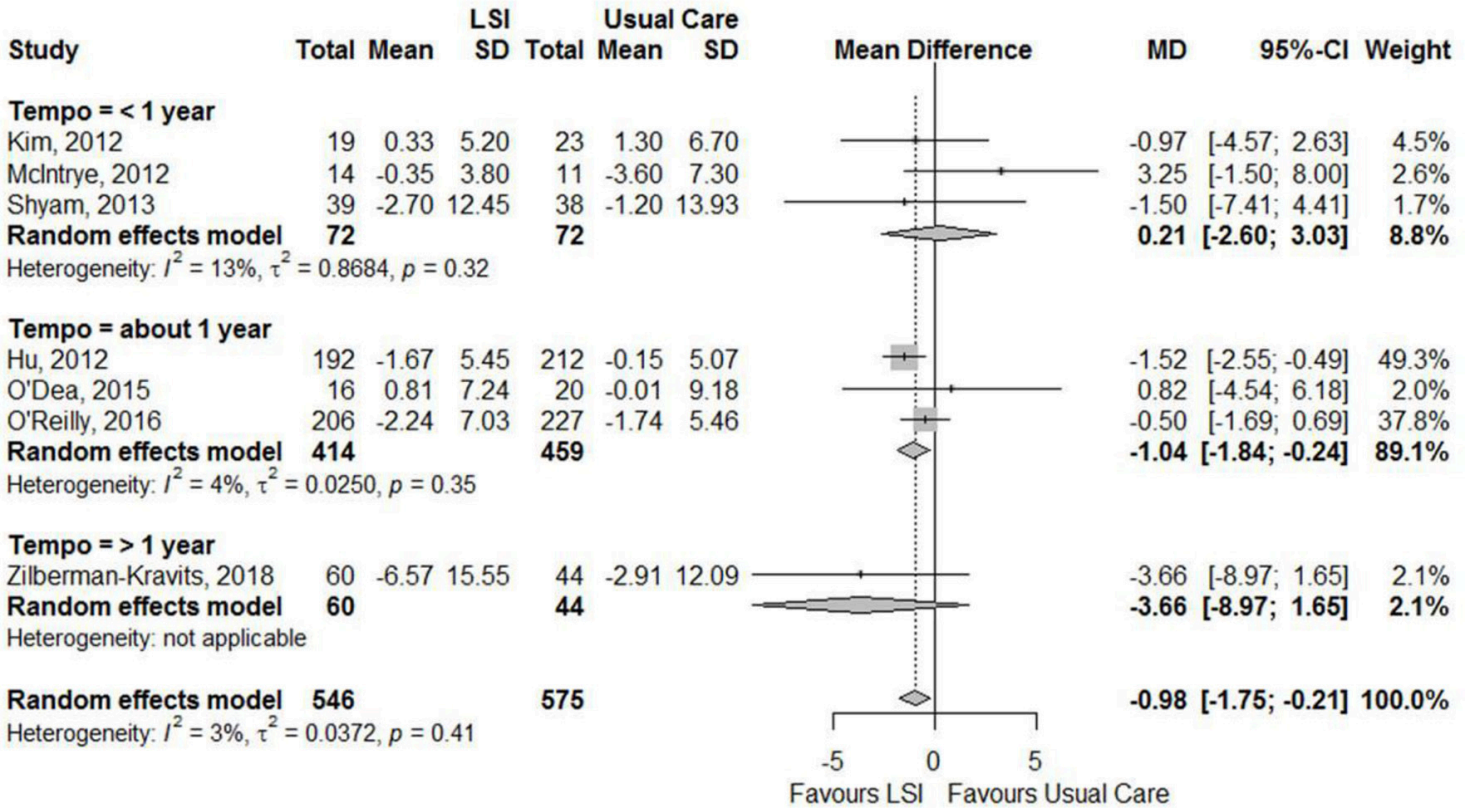
Meta-analysis of the effects of lifestyle interventions (LSI) in waist circumference change (cm) from baseline to the end of follow up, according to the duration of follow-up.

Figures 6, 7 showed that the life style intervention had a moderate statistically significant greater reductions in mean weight (MD = −1.07; 95%CI: −1.43; −0.72) kg and BMI (MD = −0.94; 95%CI: −1.79; −0.09) kg/m^2^, respectively, effects being larger with longer follow-up. Figure 8 also show a statistic significant greater reduction in waist circumference (MD = −0.98; 95%CI: −1.75; −0.21) cm, also larger with longer follow-up.

## Discussion

Evidence here summarized reveal that lifestyle changes started after a pregnancy complicated by GDM produce a 25% (RR = 0.75; 95%CI: 0.55–1.03) reduction in diabetes risk which reaches borderline statistical significance. Effects appeared to be larger when the interventions were initiated within 6 months after birth (RR = 0.61; 95%CI: 0.40–0.94; p for subgroup comparison = 0.33). We found small but statistically significant reductions in weight, BMI and waist circumference, particularly with longer periods of intervention. In contrast, we found no change with intervention for final fasting or 2 h glucose values.

The only previous meta-analysis reporting effects on diabetes incidence among women with recent gestational diabetes (26) included four of the eight trials here summarized. It did not report relative risks but found an absolute risk difference of (RD = −5.02%; 95%CI: −9.24; −0.80), consistent with the size of the risk reduction we found. With regard to weight changes, the previous meta-analysis (27) found a similar difference mean weight reduction (MD = −1.06; 95%CI: −1.68; −0.44) kg. We found no meta-analysis reporting effects on BMI, waist circumference, 2h glucose during an oral glucose tolerance test or HbA_1C_. The only one reporting a summarized effect on fasting plasma glucose, like ours did not find a statistically significant difference in reduction (MD = −0.05; 95%CI: −0.21; 0.11 mmol/L).

The fact that reductions in incidence here reported were somewhat greater when the intervention initiated sooner after birth (RR = 0.61 vs. 1.00; *p* = 0.11) may reflect stronger motivation to initiate lifestyle changes when women are closer to their GDM treatment during pregnancy. However, the number of studies initiating later is small to reach a conclusion. We have no explanation for the small size of changes in mean glucose values, but as numbers are not large, it is possible that outliers in glucose values, once diabetes developed, could influence these glucose means. Additionally, heterogeneity across studies for these outcomes was large.

We found a consistently greater effect in studies with longer follow-up across the three anthropometric measures. In these studies, the period of intervention was also of greater duration, which suggests the importance of maintaining support for lifestyle changes for a longer period, particularly given the women's frequently overwhelming tasks of motherhood. Of note also, since breastfeeding is often being performed during the post-partum period, weight loss recommended is usually small, thus requiring a longer period than the usual weight loss programs to reach weight loss goals.

There are several ongoing trials which may complete data collection and publish their results in the next three to 4 years (53–56). Up to now, this is the most comprehensive summary reporting on the feasibility and effectiveness of lifestyle modifications soon after birth of mothers with gestational diabetes. Compared to the only previous meta-analysis reporting diabetes as an outcome (26), we have increased the number of studies involved, as well as the scope of the outcomes assessed.

Although effects are small, benefits are clinically relevant, since seemingly minimal changes in anthropometric measures over a short period translate into a 25% risk reduction of diabetes in women who are, on average, only 30 years old. We hope that these ongoing trials of longer duration and with greater support for lifestyle changes will produce larger effects, perhaps with results approaching the relative risk reduction of 53% found in *post-hoc* analyses focusing on women with previous gestational diabetes (47), treated about 9 to 10 years after the target pregnancy in the similarly more robust and longer DPP study.

Our study has strengths and limitations. First, the number of women randomized (1647) and the number of events (180) are still small, resulting in only borderline statistical significance. Of note however, funnel plot and Egger test indicated small chance of publication bias. The effect of 25% reduction in the incidence of diabetes is small but potentially clinical relevant. As suggested by the absolute risk difference we found, 4%, the number needed to treat is 25 women, in other words, we need to treat 25 women with GDM at postpartum with similar interventions to prevent one case of diabetes. Finally, the quality of most studies included in this review is not high and sample size often limited to less than 70 women. These limitations highlight the need for further studies to provide more accurate summary results.

In conclusion, our comprehensive meta-analysis suggests an effect of lifestyle intervention after a pregnancy complicated by gestational diabetes. The effect is smaller than those of the classic studies of lifestyle intervention to prevent diabetes in older subjects when offered more intensive interventions. Nonetheless, the benefits here reported for younger women with previous GDM suggest that interventions to prevent diabetes are feasible and may have potential clinical. Additional studies are needed to further tailor the delivery of lifestyle interventions to this particular period of life and to improve the quality of the evidence for their effectiveness when offered to women with GDM after pregnancy.

## Author contributions

MS, PG, and WC designed the study. DS, GL, PG, and RM contributed to the literature search and data extraction. PG and PZ performed data analyses. BD, MS, PG, PZ, and WC participated in the interpretation, writing, and proofreading of the manuscript.

### Conflict of interest statement

The authors declare that the research was conducted in the absence of any commercial or financial relationships that could be construed as a potential conflict of interest.

## References

[1] International Diabetes Federation IDF Diabetes Atlas, 8th ed Brussels: International Diabetes Federation (2017). Available online at: http://www.diabetesatlas.org

[2] NCD Risk Factor Collaboration (NCD-RisC) Worldwide trends in diabetes since 1980: a pooled analysis of 751 population-based studies with 4.4 million participants. Lancet (2016) 387:1513–30. 10.1016/S0140-6736(16)00618-8 27061677 PMC5081106

[3] Pan XR Li GW Hu YH Wang JX Yang WY An ZX. Effects of diet and exercise in preventing NIDDM in people with impaired glucose tolerance. The Da Qing IGT and Diabetes Study. Diabetes Care (1997) 20:537–44. 9096977 10.2337/diacare.20.4.537

[4] Li G Zhang P Wang J Gregg EW Yang W Gong Q. The long-term effect of lifestyle interventions to prevent diabetes in the China Da Qing Diabetes Prevention Study: a 20-year follow-up study. Lancet (2008) 371:1783–9. 10.1016/S0140-6736(08)60766-7 18502303

[5] Tuomilehto J Lindström J Eriksson JG Valle TT Hämäläinen H Ilanne-Parikka P. Prevention of type 2 diabetes mellitus by changes in lifestyle among subjects with impaired glucose tolerance. N Engl J Med. (2001) 344:1343–50. 10.1056/NEJM200105033441801 11333990

[6] Knowler WC Barrett-Connor E Fowler SE Hamman RF Lachin JM Walker EA Reduction in the incidence of type 2 diabetes with lifestyle intervention or metformin. N Engl J Med. (2002) 346:393–403. 10.1056/NEJMoa012512 11832527 PMC1370926

[7] Haw JS Galaviz KI Straus AN Kowalski AJ Magee MJ Weber MB. Long-term sustainability of diabetes prevention approaches: a systematic review and meta-analysis of randomized clinical trials. JAMA Intern Med. (2017) 177:1808–17. 10.1001/jamainternmed.2017.6040 29114778 PMC5820728

[8] Mayer-Davis EJ Lawrence JM Dabelea D Divers J Isom S Dolan L. Incidence trends of type 1 and type 2 diabetes among youths, 2002-2012. N Engl J Med. (2017) 376:1419–29. 10.1056/NEJMoa1610187 28402773 PMC5592722

[9] Dabelea D Stafford JM Mayer-Davis EJ D'Agostino R Dolan L Imperatore G. Association of type 1 diabetes vs type 2 diabetes diagnosed during childhood and adolescence with complications during teenage years and young adulthood. JAMA (2017) 317:825–35. 10.1001/jama.2017.0686 28245334 PMC5483855

[10] Tobias DK Stuart JJ Li S Chavarro J Rimm EB Rich-Edwards J. Association of history of gestational diabetes with long-term cardiovascular disease risk in a large prospective cohort of US women. JAMA Intern Med. (2017) 177:1735–42. 10.1001/jamainternmed.2017.2790 29049820 PMC5820722

[11] Wein P Beischer N Harris C Permezel M. A trial of simple versus intensified dietary modification for prevention of progression to diabetes mellitus in women with impaired glucose tolerance. Aust N Z J Obstet Gynaecol. (1999) 39:162–6. 10.1111/j.1479-828X.1999.tb03363.x 10755770

[12] Kim C Draska M Hess ML Wilson EJ Richardson CR. A web-based pedometer programme in women with a recent history of gestational diabetes. Diabet Med. (2012) 29:278–83. 10.1111/j.1464-5491.2011.03415.x 21838764 PMC4139030

[13] Nicklas JM Zera CA England LJ Rosner BA Horton E Levkoff SE. A web-based lifestyle intervention for women with recent gestational diabetes mellitus: a randomized controlled trial. Obstet Gynecol. (2014) 124:563–70. 10.1097/AOG.0000000000000420 25162257 PMC4401073

[14] Peacock AS Bogossian FE Wilkinson SA Gibbons KS Kim C McIntyre HD. A randomised controlled trial to delay or prevent type 2 diabetes after gestational diabetes: walking for exercise and nutrition to prevent diabetes for you. Int J Endocrinol. (2015) 2015:423717. 10.1155/2015/423717 26089886 PMC4452189

[15] McIntyre HD Peacock A Miller YD Koh D Marshall AL. Pilot study of an individualised early postpartum intervention to increase physical activity in women with previous gestational diabetes. Int J Endocrinol. (2012) 2012:892019. 10.1155/2012/892019 22548057 PMC3324899

[16] O'Reilly SL Dunbar JA Versace V Janus E Best JD Carter R. Mothers after Gestational Diabetes in Australia (MAGDA): a randomised controlled trial of a postnatal diabetes prevention program. PLoS Med. (2016) 13:e1002092. 10.1371/journal.pmed.1002092 27459502 PMC4961439

[17] Shek NWM Ngai CSW Lee CP Chan JYC Lao TTH. Lifestyle modifications in the development of diabetes mellitus and metabolic syndrome in Chinese women who had gestational diabetes mellitus: a randomized interventional trial. Arch Gynecol Obstet. (2014) 289:319–27. 10.1007/s00404-013-2971-0 23897066

[18] Wen JW Effects of a Lifestyle Intervention on Glycometabolism in Women Having Gestational Diabetes Mellitus and Impaired Glucose Tolerance After Delivery. Beijing: School of Nursing, PeKing Union Medical College (2011).

[19] Hu G Tian H Zhang F Liu H Zhang C Zhang S. Tianjin Gestational Diabetes Mellitus Prevention Program: study design, methods, and 1-year interim report on the feasibility of lifestyle intervention program. Diabetes Res Clin Pract. (2012) 98:508–17. 10.1016/j.diabres.2012.09.015 23010556

[20] Yu X Wu X Zhang Y Mao Y The effects of lifestyle intervention on insulin resistance and islet β cell function in gestational diabetes patients with postpartum impaired glucose regulation. Chin J Prev Control Chronic Non-Commun Dis. (2012) 560–2. 10.16386/j.cjpccd.issn.1004-6194.2012.05.037

[21] Pérez-Ferre N Del Valle L Torrejón MJ Barca I Calvo MI Matía P. Diabetes mellitus and abnormal glucose tolerance development after gestational diabetes: A three-year, prospective, randomized, clinical-based, Mediterranean lifestyle interventional study with parallel groups. Clin Nutr. (2015) 34:579–85. 10.1016/j.clnu.2014.09.005 25262459

[22] Shyam S Arshad F Abdul Ghani R Wahab NA Safii NS Nisak MYB. Low glycaemic index diets improve glucose tolerance and body weight in women with previous history of gestational diabetes: a six months randomized trial. Nutr J. (2013) 12:68. 10.1186/1475-2891-12-68 23705645 PMC3671161

[23] Zilberman-Kravits D Meyerstein N Abu-Rabia Y Wiznitzer A Harman-Boehm I. The impact of a cultural lifestyle intervention on metabolic parameters after gestational diabetes mellitus a randomized controlled trial. Matern Child Health J (2018) 22:803–11. 10.1007/s10995-018-2450-0 29411251

[24] O'Dea A Tierney M McGuire BE Newell J Glynn LG Gibson I. Can the Onset of type 2 diabetes be delayed by a group-based lifestyle intervention in women with prediabetes following gestational diabetes mellitus (GDM)? findings from a randomized control mixed methods trial. J Diabetes Res. (2015) 2015:798460. 10.1155/2015/798460 26347894 PMC4546980

[25] Cheung NW Smith BJ van der Ploeg HP Cinnadaio N Bauman A. A pilot structured behavioural intervention trial to increase physical activity among women with recent gestational diabetes. Diabetes Res Clin Pract. (2011) 92:e27–29. 10.1016/j.diabres.2011.01.013 21316788

[26] Pedersen ALW Terkildsen Maindal H Juul L. How to prevent type 2 diabetes in women with previous gestational diabetes? A systematic review of behavioural interventions. Prim Care Diabetes (2017) 11:403–13. 10.1016/j.pcd.2017.05.002 28601549

[27] Gilinsky AS Kirk AF Hughes AR Lindsay RS. Lifestyle interventions for type 2 diabetes prevention in women with prior gestational diabetes: a systematic review and meta-analysis of behavioural, anthropometric and metabolic outcomes. Prev Med Rep. (2015) 2:448–61. 10.1016/j.pmedr.2015.05.009 26844102 PMC4721374

[28] Guo J Chen JL Whittemore R Whitaker E. Postpartum lifestyle interventions to prevent type 2 diabetes among women with history of gestational diabetes: a systematic review of randomized clinical trials. J Womens Health (2016) 25:38–49. 10.1089/jwh.2015.5262 26700931

[29] Liberati A Altman DG Tetzlaff J Mulrow C Gøtzsche PC Ioannidis JPA. The PRISMA statement for reporting systematic reviews and meta-analyses of studies that evaluate health care interventions: explanation and elaboration. J Clin Epidemiol. (2009) 62:e1–34. 10.1016/j.jclinepi.2009.06.006 19631507

[30] Mukerji G McTavish S Glenn A Delos-Reyes F Price J Wu W. An innovative home-based cardiovascular lifestyle prevention program for women with recent gestational diabetes: a pilot feasibility study. Can J Diabetes (2015) 39:445–50. 10.1016/j.jcjd.2015.08.002 26482886

[31] Philis-Tsimikas A Fortmann AL Dharkar-Surber S Euyoque JA Ruiz M Schultz J. Dulce Mothers: an intervention to reduce diabetes and cardiovascular risk in Latinas after gestational diabetes. Transl Behav Med. (2014) 4:18–25. 10.1007/s13142-014-0253-4 24653773 PMC3958598

[32] Brazeau AS Leong A Meltzer SJ Cruz R DaCosta D Hendrickson-Nelson M. Group-based activities with on-site childcare and online support improve glucose tolerance in women within 5 years of gestational diabetes pregnancy. Cardiovasc Diabetol. (2014) 13:104. 10.1186/1475-2840-13-104 24981579 PMC4227099

[33] Ferrara A Ehrlich SF Feng J Quesenberry CP Moore SD Hedderson MM Postpartum weight loss is associated with improved glucose and insulin homeostasis in women with a history of gestational diabetes (GDM). Diabetes (2012) 61:A339.

[34] Liu H Wang L Zhang S Leng J Li N Li W. One-year weight losses in the Tianjin Gestational Diabetes Mellitus Prevention Programme: a randomized clinical trial. Diabetes Obes Metab. (2018) 20:1246–55. 10.1111/dom.13225 29360237 PMC5899932

[35] Liu H Wang L Zhang S Leng J Li N Li W 1 year weight losses in the Tianjin Gestational Diabetes Mellitus Prevention Program: a randomised trial. Lancet Diabetes Endocrinol. (2016) 4:S11 10.1016/S2213-8587(16)30366-7 PMC5899932 29360237

[36] McCance DR Draffin C Patterson CC Francis L Irwin J McConnell M Postnatal lifestyle intervention for overweight women with previous gestational diabetes mellitus (PAIGE): a pilot randomised controlled trial. Ir J Med Sci. (2016) 185:S401 10.1210/jc.2017-02654 29762737

[37] McManus R Miller D Mottola M Giroux I Donovan L. Translating healthy living messages to postpartum women and their partners after gestational diabetes (GDM): body habitus, A1C, lifestyle habits, and program engagement results from the families defeating diabetes (FDD) randomized trial. Am J Health Promot. (2017) 32:1438–46. 10.1177/0890117117738210 29108443

[38] Reinhardt JA van der Ploeg HP Grzegrzulka R Timperley JG. lmplementing lifestyle change through phone-based motivational interviewing in rural-based women with previous gestational diabetes mellitus. Health Promot J Austr. (2012) 23:5–9. 10.1071/HE12005 22730940

[39] Smith BJ Cinnadaio N Cheung NW Bauman A Tapsell LC van der Ploeg HP. Investigation of a lifestyle change strategy for high-risk women with a history of gestational diabetes. Diabetes Res Clin Pract. (2014) 106:e60-63. 10.1016/j.diabres.2014.09.035 25451910

[40] Stuebe AM Bonuck K Adatorwovor R Schwartz TA Berry DC. A Cluster randomized trial of tailored breastfeeding support for women with gestational diabetes. Breastfeed Med. (2016) 11:504–13. 10.1089/bfm.2016.0069 27782758 PMC5165668

[41] Athavale P Thomas M Delgadillo-Duenas AT Leong K Najmabadi A Harleman E. Linking high risk postpartum women with a technology enabled health coaching program to reduce diabetes risk and improve wellbeing: program description, case studies, and recommendations for community health coaching programs. J Diabetes Res. (2016) 2016:4353956. 10.1155/2016/4353956 27830157 PMC5088315

[42] Brown SD Guterman J Gordon N Tsai AL Hedderson MM Ferrara A Evaluating a postpartum diabetes prevention program: the gestational diabetes' effects on moms (GEM) trial. Diabetes (2017) 66:A216.

[43] Ferrara A Hedderson MM Brown SD Albright CL Ehrlich SF Tsai A-L. The comparative effectiveness of diabetes prevention strategies to reduce postpartum weight retention in women with gestational diabetes mellitus: the gestational diabetes' effects on moms (GEM) cluster randomized controlled trial. Diabetes Care (2016) 39:65–74. 10.2337/dc15-1254 26657945 PMC4686847

[44] Ferrara A Hedderson MM Albright CL Brown SD Ehrlich SF Mevi AA Reduced postpartum weight retention with a dpp-derived lifestyle intervention: the gestational diabetes' effects on moms (GEM) cluster randomized trial. Diabetes (2014) 63:A94–5.

[45] Ferrara A Hedderson MM Albright CL Ehrlich SF Quesenberry CP Peng T. A pregnancy and postpartum lifestyle intervention in women with gestational diabetes mellitus reduces diabetes risk factors: a feasibility randomized control trial. Diabetes Care (2011) 34:1519–25. 10.2337/dc10-2221 21540430 PMC3120183

[46] Jelsma JGM van Poppel MN M Smith BJ Cinnadaio N Bauman A Tapsell L. Changing psychosocial determinants of physical activity and diet in women with a history of gestational diabetes mellitus. Diabetes Metab Res Rev. (2018) 34:e2942. 10.1002/dmrr.2942 28843034

[47] Ratner RE Christophi CA Metzger BE Dabelea D Bennett PH Pi-Sunyer X. Prevention of diabetes in women with a history of gestational diabetes: effects of metformin and lifestyle interventions. J Clin Endocrinol Metab. (2008) 93:4774–9. 10.1210/jc.2008-0772 18826999 PMC2626441

[48] Aroda VR Christophi CA Edelstein SL Zhang P Herman WH Barrett-Connor E. The effect of lifestyle intervention and metformin on preventing or delaying diabetes among women with and without gestational diabetes: the Diabetes Prevention Program outcomes study 10-year follow-up. J Clin Endocrinol Metab. (2015) 100:1646–53. 10.1210/jc.2014-3761 25706240 PMC4399293

[49] Huvinen H Koivusalo S StachLempinen B Kautiainen H Eriksson J Effects of a lifestyle intervention during pregnancy and 1-year postpartum -results from the RADIEL study. Gynecol Endocrinol. (2016) 32:161.26494397

[50] Huvinen E Koivusalo SB Meinilä J Valkama A Tiitinen A Rönö K. Effects of a lifestyle intervention during pregnancy and first postpartum year: findings from the RADIEL Study. J Clin Endocrinol Metab. (2018) 103:1669–77. 10.1210/jc.2017-02477 29409025

[51] Shyam S Fatimah A Rohana AG Norasyikin AW Karuthan C Shanita N Lowering dietary glycaemic index through nutrition education among Malaysian women with a history of gestational diabetes mellitus. Malays J Nutr. (2013) 19:9–24. 24800381

[52] Ghani RA Shyam S Arshad F Wahab NA Chinna K Safii NS. The influence of fasting insulin level in post-gestational diabetes mellitus women receiving low-glycaemic-index diets. Nutr Diabetes (2014) 4:e107. 10.1038/nutd.2014.5 24535618 PMC3940829

[53] Schmidt MI Duncan BB Castilhos C Wendland EM Hallal PC Schaan BD. Lifestyle INtervention for Diabetes prevention After pregnancy (LINDA-Brasil): study protocol for a multicenter randomized controlled trial. BMC Pregnancy Childbirth (2016) 16:68. 10.1186/s12884-016-0851-x 27029489 PMC4812654

[54] Chasan-Taber L Marcus BH Rosal MC Tucker KL Hartman SJ Pekow P. Estudio Parto: postpartum diabetes prevention program for hispanic women with abnormal glucose tolerance in pregnancy: a randomised controlled trial - study protocol. BMC Pregnancy Childbirth (2014) 14:100. 10.1186/1471-2393-14-100 24606590 PMC3975296

[55] Clinical Trials,.gov. Identifier NCT03559621. Melinda Pilot Study (MELINDA) Bethesda, MD: National Library of Medicine (2018). Available online at: https://clinicaltrials.gov/ct2/show/NCT03559621?term=NCT03559621&rank=1

[56] ClinicalTrials.gov. Identifier NCT02744300. Balance After Baby Intervention for Women with Recent Gestational Diabetes (BABI2) Bethesda, MD: National Library of Medicine (2016). Available online at: https://clinicaltrials.gov/ct2/show/NCT02744300?term=NCT02744300&rank=1

